# Internet searching and stock price informativeness: Evidence from Google withdrawal in China

**DOI:** 10.1371/journal.pone.0297160

**Published:** 2024-03-13

**Authors:** Shuxian Li, Xinheng Liu

**Affiliations:** 1 School of Business, Sun Yat-Sen University, Guangzhou, Guangdong Province, China; 2 School of Economics and Management, Changsha University of Science & Technology, Changsha, Hunan Province, China; University of Baltistan, PAKISTAN

## Abstract

We analyze whether and how internet searching impacts stock price informativeness. Using the 2010 Google withdrawal in China as a quasi-natural experiment, we establish a causal effect between internet searching and stock price informativeness using a difference-in-difference framework. We find that firms with higher Google search volume experience a 10% decrease in stock price informativeness after the Google withdrawal. The negative effect of the Google withdrawal on stock price informativeness is pronounced in firms with more retail investors, larger state-ownership, and poor analysts’ earnings forecasts. Our results suggest that retail investors can benefit from internet searching to collect and process firm-specific information more efficiently.

## 1. Introduction

Stock markets are vital information hubs for modern economies, and they serve not just as a shadow for firm operations but also have a price discovery function [1, 2]. When making business decision-making, managers can acquire alternative information from stock prices [1] and improve managerial efficiency [3]. Then, it can promote firms’ productivity eventually [4]. Therefore, looking for the determinate factors for stock price informativeness has important policy implications, specifically in enhancing the feedback efficiency of the stock market. In this paper, we attempt to investigate a question that has not yet been discussed in the existing literature whether and how internet searching affects stock price informativeness under the background of the widespread use of the internet.

It is well known that the widespread use of the internet allows people to access external information without leaving their homes, significantly reducing the cost of information acquisition [5]. This trend is particularly pronounced in stock markets with more retail investors. What’s more, compared with institutional investors, internet searching is the less costly way for retail investors to access firms’ information and help them make stock buying and selling decisions [6]. In addition, other scholars find that internet searching can enhance the efficiency of capital markets by alleviating firms’ information bias among retail investors [3] and effectively predicting stock price return or volatility [7, 8]. Therefore, building on these arguments, we hypothesize that internet searching could promote stock prices incorporate more firms’ specific information. However, two identification challenges need to be addressed to examine the causal effect of internet searching on stock price informativeness. First, the relationship between investors’ internet searching and stock prices movement can be driven by unobservable factors. Second, in the developed financial market, institutional investors have absolute pricing power, and retail investors who search information online only exert a more negligible effect on trading activity at the market level.

To effectively address the two issues of endogeneity and appropriate sample selection, we employed an exogenous event—Google unexpected withdrawal from mainland China in 2010—to conduct empirical analysis. The reasons are as follows. First and foremost, Google suddenly shut down its search business in China as it failed to persuade the Chinese government to relax its search results censorship. Although Baidu has become the largest search engine in China after Google withdrawal, its’ search results can be considered to be manipulated [9]. Secondly, retail investors play a vital role in the Chinese stock market. In 2017, retail investors trading accounted for 80% of the total transactions (See http://www.sse.com.cn/aboutus/research/special/c/4498328.pdf), which could further increase the credibility of our empirical results.

Therefore, we conducted an empirical analysis from Chinese Stock Market & Accounting Research (CSMAR) databases for Chinese listed companies from 2007 to 2012. For the key explanatory variable, namely the stock price informativeness, we adopted two methods in accordance with the practices of previous studies [10, 11] to reduce measurement bias concerns. Furthermore, given the substantial variation in search volume among firms before the withdrawal from Google, we can use a difference-in-differences (DID) method to investigate the impact of internet searching on the stock price informativeness. Our empirical findings show that, compared with the control firms, stock price informativeness for treated firms decreased by about 10% after Google withdrawal, which suggests that internet searching can help retail investors obtain specific information and improve stock market pricing. In addition, cross-sectional tests reveal that the effect is more pronounced in firms with more retail investors, larger state-ownership, and poor analysts’ earning forecast accuracy. We can further confirm that internet searching can alleviate the information asymmetry for retail investors and improve stock market informativeness.

The contributions of this paper have two dimensions. First, a growing number of researchers have paid attention to the determinants of stock price informativeness, such as information disclosure [12], nature of control shareholder [13], legal system [14] and so on. Complementary, we identify the causal effect of internet searching on the stock price informativeness from retail investor information acquisition. Secondly, we contribute to the literature on the capital market effect of the internet. Extensive research mainly focuses on the role of social media in the capital market [15]. Our paper is close to Xu et al. [3], who found that internet searching can significantly alleviate stock price crash risks. Distinctively, we examine how individual investors’ search for information online affects the stock price informativeness.

The remainder of this paper is organized as follows: Section 2 presents our empirical design and data description, while Sections 3 and 4 present the empirical results. Section 5 discusses our findings, and Section 6 concludes the paper.

## 2. Data and methodology

### 2.1 Data selection

The sample period chosen is crucial for the reliability of the empirical results. Considering the fact that Google withdrawal occurred in 2010 and the new accounting standards were implemented in 2007, we chose a sample period from 2007 to 2014 to balance the period before and after the Google withdrawal. We began to obtain financial and stock return data from the Chinese Stock Market & Accounting Research (CSMAR) databases, which include all Chinese A-share listed companies. Then, we matched these data by ticker symbol to the Google search volume index data provided by Xu et al. [3] (The data can be download from the website: http://jfe.rochester.edu/Xu_Xuan_Zheng_data.xlsx) and removed the unmatched data. We further drop the companies with the following characteristics in the process of sample refinements. 1) Companies in the financial industry and special treatment (ST) and particular transfer (PT) companies; 2) Companies with missing financial data; 3) Companies listed in 2010 and afterward. To rule out the effect of outliers, we winsorize all continuous firms’ variables at 1% and 99% levels. Finally, our sample contains 9801 firm-year observations.

### 2.2 Measuring stock price informativeness

According to the suggestions by Roll [16], if a stock incorporates relatively more firm-specific information, it implies that its price has less co-move with other stocks. In other words, the stock price synchronicity with the market is lower. Therefore, to measure the stock price informativeness, the first step is to calculate the extent to which the stock price can be explained by the market index, i.e., *R*^*2*^. Following Morck et al. [10], we can obtain firm-level synchronicity measured by the *R*^*2*^, for each stock in each year, by running the following regression:

$$r_{i,j,t}=\alpha_{j}+\beta_{1}*r_{m,t}+\beta_{2}*r_{j,t}+\varepsilon_{i,j,t},$$ (1)

where the subscripts i, j, and t represent firm, industry, and year, respectively. *r_i,j,t_* is the stock return for firm i, in industry j and at week t. *r_m,t_* is the value weighted market return on week t. *r_j,t_* is the value weighted industry return on week t. The weights are calculated using firms’ market capitalization. When calculating the market and industry return for firm i, firms i is eliminated to avoid the spurious problem between industry return and firm return [4]. Note that the industry classification is based on the 2012 edition of the CSRC Guidelines for Industry Classification of Listed Companies. Moreover, Model (1) is estimated for each stock in each year but requires no less than 26 weekly observations.

As an alternative measurement, we add the lagged market and industry returns values into the model (1) to control the lagging effects [11]. The regression model is as follows:

$$r_{i,j,t}=\alpha_{j}+\beta_{1}*r_{m,t}+\beta_{2}*r_{j,t}+\beta_{3}*r_{m,t-1}+\beta_{4}*r_{j,t-1}+\varepsilon_{i,j,t},$$ (2)

where *r*_*m,t*−1_ is the value weighted market return on week t-1.*r*_*j,t*−1_ is the value weighted industry return on week t-1. Meanwhile, we conduct the estimation of Model (2) like Model (1), then we can get another *R*^*2*^ at the firm-year level. Therefore, we can use *R*^*2*^ obtained from the regression of Model (1) and Model (2) to construct the stock price informativeness as follows:

$$SPI_{i,t}=ln\left(\frac{1-R_{i,t}^{2}}{R_{i,t}^{2}}\right),$$ (3)

The larger the *SPI*, the more firm-specific information contained in the stock price movement, and the higher the stock price informativeness. For the sake of differentiation, we use *SPI1* and *SPI2* to represent the stock price informativeness computed with R2 estimated from Model (1) and Model (2), respectively.

### 2.3 DID specification

Internet search engines are one of the primary channels for investors to gain timely access to information about companies. Google stands out as a representative company in the field of internet search engines, leading in terms of information coverage and search quality compared to other enterprises. As a multinational technology company, Google Search entered the Chinese Mainland market in April 2006 and has steadily increased its market share since then. On the eve of the decision to withdraw from China in 2010, Google and Baidu accounted for 30% and 58% of the market share, respectively, in the Chinese search engine market (See https://www.technologyreview.com/2018/12/19/138307/how-google-took-on-china-and-lost/). Noticeably, Baidu’s market share grows rapidly to 80% after Google announced its withdrawal from mainland China. Although Baidu occupies the vast majority of mainland China’s search engine market share, it is often regarded as manipulating search results, making it difficult for users to seek out useful information [9]. More precisely, Google withdrawal from mainland China began on January 13, 2010, when the company announced a new program to apply a new technology to combat hacker attacks. However, during months of negotiations regarding freedom of information, censorship, and cybersecurity, Google’s program failed to reach an agreement with the Chinese government. On early March 23, Google shut down its servers in mainland China. Although internet users in mainland China can still log in to Google search engine by circumventing the firewall, many sensitive websites are still filtered out [17], which greatly increases the cost of obtaining valuable information from the internet for them. Anticipating that Google withdrawal may heighten the challenge for investors in accessing detailed company information, subsequently reducing the stock price informativeness.

Given the aforementioned facts, the event of Google withdrawal from China unfolded rapidly, materializing within a mere three months, catching nearly everyone by surprise. Hence, it can be characterized as a quasi-natural experiment. We, thus, use the 2010 Google withdrawal event as a quasi-natural experiment and employ a Difference-in-Differences (DID) model as follows to investigate the impact of internet searching on stock price informativeness:

$$SPI1_{i,t}/SPI2_{i,t}=\alpha +\beta *TREAT_{i}*POST_{t}+Control_{i,t}+FIRM_{i}+YEAR_{t}+\varepsilon_{i,t},$$ (4)

where *SPI1*_*i*,*t*_ and *SPI2*_*i*,*t*_ is the stock price informativeness for firms i at year t, calculated based on *R*^*2*^ for model(1) and (2) respectively. *TREAT*_*i*_ is the treatment group variable, which equals 1 for firms whose stock tickers have a higher Google search volume index(SVI) than the sample median in 2009, and 0 otherwise. The SVI of a ticker symbol can often represent investors’ search for relevant information about listed companies [18]. A higher SVI indicates that investors rely more on the Google search engine to obtain firm-specific information, while it will become difficult to obtain such information after Google withdrawal. To conduct a difference-in-differences estimation, we also define another dummy variable *POST*_*i*_, which indicates the post-treatment variable at year t, which equals 1 for firms in 2010–2014, and 0 otherwise.

We also control for a battery of firm-level variables that prior studies have shown can have a basic impact on stock price informativeness [19]. These variables include the size of market value of equity (*MVE*), market-to-book ratio (*MTB*), institutional shareholding ratio (*INST*), and trading turnover ratio (*TURN*). These variables are strongly related to corporate governance or investor attention and thus may be a omitted variables in the effect of internet searching on the stock price informativeness. We also control for the stock return skewness (*SKEW*) and capital market beta (*BETA*), as the stock price informativeness may be influenced by these two market variables(Jin and Myers, 2006; Li et al., 2014). Additionally, we also include the control variables that may help investors obtain information, including analyst coverage (*ANALYST*), media coverage (*MEDIA*), and the Baidu search index (*BAIDU*). To some extent, analysts, media, and Baidu searches can serve as alternative channels for information retrieval compared to Google search. Thus, these indicators may simultaneously influence Google’s search volume and stock price informativeness, serving as important confounding factors in the Google withdrawal effect. All variables are defined in Table A1 of S1 Appendix. *FIRM* and *YEAR* is firm and year fixed effects, respectively. The coefficients estimation also clustering at the firm level to control serial correlations in the error term.

Table 1 provides descriptive statistics of our sample. The mean (standard deviation) value of *SPI1* and *SPI2* is equal to 0.181 (0.812) and 0.045 (0.752), respectively, which suggests that there are considerable differences in stock price informativeness across firms or over time. These descriptive statistics differ somewhat from Ullah et al. [20] and Chen et al. [21], probably explained by the different periods chosen for the sample. Regarding explanatory variables, the treatment firms’ mean value is 0.526, implying that more than 50% of firms could be affected more by Google withdrawal, comparable to the recent work of Xu et al. [3]. The mean value of the year dummy variable *POST* is 0.650, indicating that the time before and after the shock is roughly equal. In addition, the statistics of the control variables are basically consistent with previous studies [19, 22, 23].

**Table 1:** Descriptive statistics.

	*N*	*Mean*	*Std*. *Dev*.	*p25*	*p50*	*p75*
*SPI1*	9,801	0.181	0.812	-0.382	0.131	0.672
*SPI2*	9,801	0.045	0.752	-0.473	0.009	0.524
*TREAT*	9,801	0.526	0.499	0.000	1.000	1.000
*POST*	9,801	0.650	0.477	0.000	1.000	1.000
*MVE*	9,801	22.31	0.994	21.61	22.18	22.88
*MTB*	9,801	2.028	1.493	1.034	1.566	2.475
*INST*	9,801	0.073	0.089	0.008	0.037	0.103
*TURN*	9,801	3.689	2.451	1.842	3.089	4.966
*BETA*	9,801	1.101	0.223	0.965	1.106	1.241
*SKEW*	9,801	0.134	0.443	-0.138	0.058	0.343
*ANALYST*	9,801	1.517	1.170	0.693	1.386	2.485
*MEDIA*	9,801	2.983	1.549	2.303	3.135	3.970
*BAIDU*	9,801	5.123	2.193	5.313	5.897	6.332

## 3. Main results

### 3.1 The effect of internet searching on the stock price informativeness

Table 2 presents the results of the estimating Model (4). In Columns (1) and (3), Model (4) is estimated without any control variable. The coefficient of TREAT*POST is negative and statistically significant at the 1% level. The preliminary indication is that Google withdrawal decreases the stock price informativeness. In Columns (2) and (4), all control variables are added, the coefficient of TREAT*POST is -0.081 and -0.076, and both are statistically significance at 1% level. This negative effect is also economically significant in that a one-standard-deviation variation in TREAT*POST could lead to the sample mean of *SPI1* and *SPI2* decreasing by 9.98% (= -0.081/0.812) and 10.11% (-0.076/0.752), respectively. Our results support the argument that internet searching could help investors facilitate information processing and promote stock market efficiency.

**Table 2:** The effect of Google withdrawal on the stock price informativeness.

	(1)	(2)	(3)	(4)
	*SPI1*	*SPI1*	*SPI2*	*SPI2*
TREAT*POST	-0.127***	-0.081***	-0.117***	-0.076***
	(0.029)	(0.027)	(0.028)	(0.025)
*MVE*		0.228***		0.224***
		(0.024)		(0.022)
*MTB*		0.063***		0.053***
		(0.008)		(0.008)
*INST*		0.576***		0.581***
		(0.134)		(0.124)
*TURN*		0.077***		0.073***
		(0.004)		(0.004)
*BETA*		-1.360***		-1.199***
		(0.043)		(0.039)
*SKEW*		0.201***		0.168***
		(0.017)		(0.016)
*ANALYST*		-0.029**		-0.030***
		(0.011)		(0.011)
*MEDIA*		0.023**		0.012
		(0.012)		(0.011)
*BAIDU*		0.016***		0.012**
		(0.005)		(0.005)
*Constant*	0.385***	-4.065***	0.226***	-4.198***
	(0.018)	(0.514)	(0.017)	(0.483)
Firm fixed effects	*Yes*	*Yes*	*Yes*	*Yes*
Year fixed effects	*Yes*	*Yes*	*Yes*	*Yes*
N	9,801	9,801	9,801	9,801
Adj. *R*^*2*^	0.331	0.475	0.325	0.458

Notes: Standard errors are robust and clustered at the firm level

***, **, * indicate significance at the 1%, 5%, and 10% levels, respectively.

### 3.2 Robustness checks

#### 1. Parallel trends assumption

To examine the dynamic effect, we augment Model (4) by replacing TREAT*POST with the interaction term between *TREAT* and the other three dummy variables respectively, which are one year before the withdrawal (*BEFORE*^*1*^), the year of withdrawal (*AFTER*^*0*^), and all the years after the withdrawal (*AFTER*^*1+*^). The results shown in Columns 1 and 2 of Table 3, the coefficient of TREAT*BEFORE^*1*^ is -0.010 and -0.004, respectively, but neither is statistically insignificant, implying no preexisting stock price informativeness trends before the Google withdrawal. Our dynamic analysis results suggest that our results are align with a causal interpretation.

**Table 3:** Test for validity of the DID model specification.

	(1)	(2)	(3)	(4)	(5)	(6)
	*SPI1*	*SPI2*	*SPI1*	*SPI2*	*SPI3*	*PIN*
TREAT*BEFORE ^*1*^	-0.010	-0.004				
	(0.034)	(0.032)				
TREAT*BEFORE ^*0*^	-0.098**	-0.085**				
	(0.038)	(0.037)				
TREAT*BEFORE ^*1+*^	-0.082**	-0.076**				
	(0.033)	(0.031)				
TREAT*POST			-0.055*	-0.055*	-0.149***	-0.132**
			(0.031)	(0.029)	(0.030)	(0.058)
*MVE*	0.229***	0.224***	0.255***	0.246***	0.244***	-0.262***
	(0.024)	(0.022)	(0.028)	(0.026)	(0.027)	(0.050)
*MTB*	0.063***	0.053***	0.066***	0.056***	0.048***	0.094***
	(0.008)	(0.008)	(0.010)	(0.010)	(0.009)	(0.016)
*INST*	0.576***	0.581***	0.441***	0.490***	0.307**	4.171***
	(0.134)	(0.125)	(0.165)	(0.154)	(0.152)	(0.255)
*TURN*	0.077***	0.073***	0.085***	0.081***	0.056***	-0.201***
	(0.004)	(0.004)	(0.005)	(0.005)	(0.005)	(0.008)
*BETA*	-1.360***	-1.199***	-1.361***	-1.199***	-0.816***	-0.605***
	(0.043)	(0.039)	(0.050)	(0.046)	(0.051)	(0.084)
*SKEW*	0.201***	0.168***	0.216***	0.182***	0.230***	-0.054*
	(0.017)	(0.016)	(0.019)	(0.018)	(0.019)	(0.030)
*ANALYST*	-0.029**	-0.030***	-0.036***	-0.036***	-0.008	-0.024
	(0.011)	(0.011)	(0.013)	(0.012)	(0.013)	(0.021)
*MEDIA*	0.023*	0.012	0.022*	0.008	0.052***	-0.099***
	(0.012)	(0.011)	(0.013)	(0.012)	(0.015)	(0.021)
*BAIDU*	0.017***	0.012**	0.018***	0.014**	0.000	-0.025**
	(0.005)	(0.005)	(0.006)	(0.006)	(0.005)	(0.011)
*Constant*	-4.073***	-4.202***	-4.701***	-4.734***	-4.971***	24.400***
	(0.515)	(0.484)	(0.606)	(0.569)	(0.569)	(1.092)
Firm fixed effects	*Yes*	*Yes*	*Yes*	*Yes*	*Yes*	*Yes*
Year fixed effects	*Yes*	*Yes*	*Yes*	*Yes*	*Yes*	*Yes*
N	9,801	9,801	7,292	7,292	9,801	9,801
Adj. *R*^*2*^	0.475	0.458	0.489	0.472	0.817	0.512

Notes: Standard errors are robust and clustered at the firm level

***, **, * indicate significance at the 1%, 5%, and 10% levels, respectively.

#### 2. Placebo test

**Fig 1:**
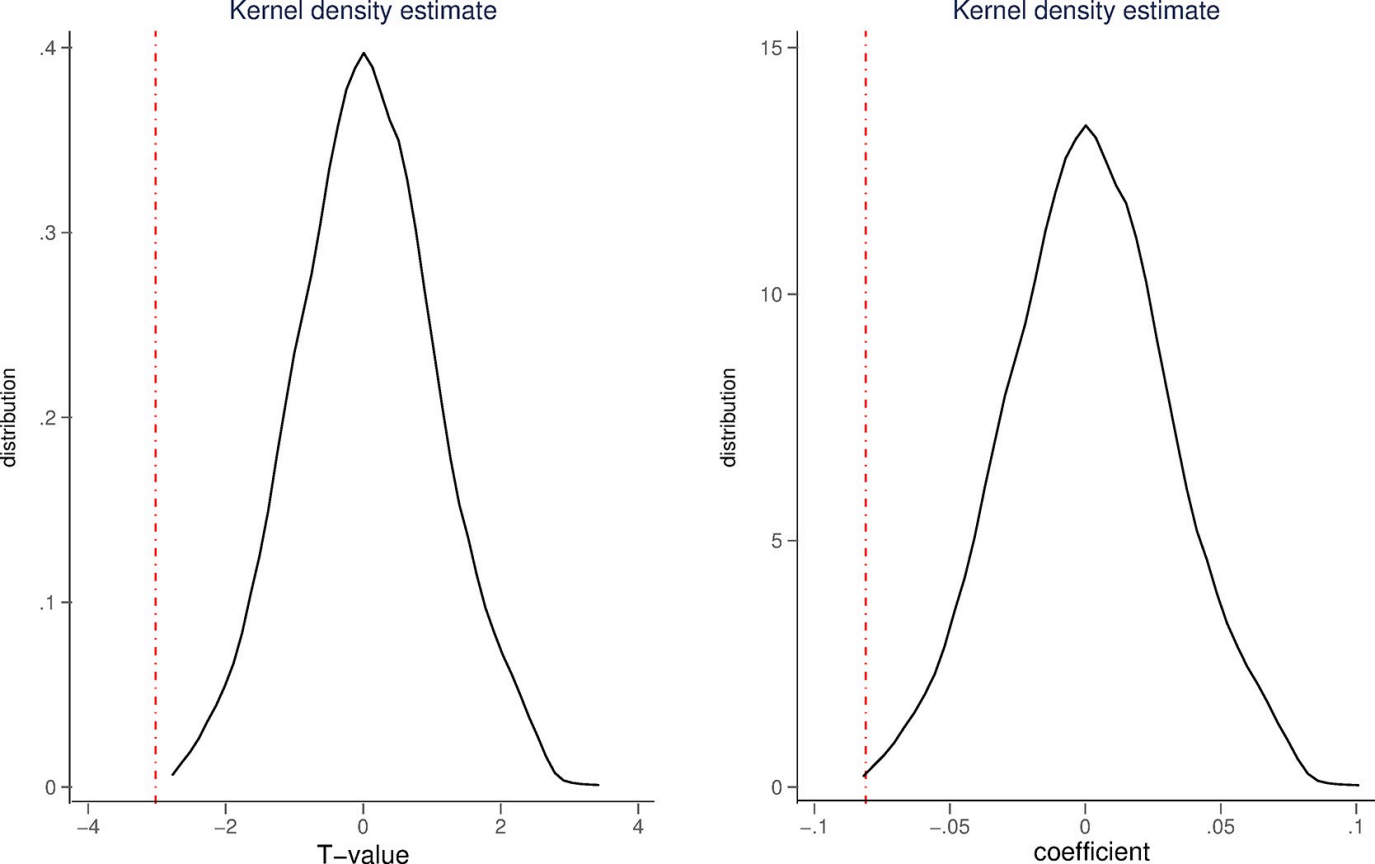
Sampling 1000 times for determining the treatment group.

One may be concerned that the treatment group is not random to assign, which could lead our baseline regression results to spurious correlations. Mitigating this concern is indispensable for identifying the causal effect of Google withdrawal on the stock price informativeness. Hence, following Chetty et al. [24], we randomly sample to determine the fake treatment group (denoted as *PSEUDO TREAT*) and perform the estimation for the baseline model with the full set of control variables. This step is repeated for1000 times. All the estimated T-values and coefficients for the interaction term of PSEUDO TREAT*POST are shown in Fig 1, respectively. Consistent with randomization, the coefficients for PSEUDO TREAT*POST have a distribution centered on 0, and approximately 90% of the absolute T-values are below 1.65 (Corresponding to 10% significance level). The true estimated coefficients and T-values for TREAT*POST (both are represented by the red line) appear as outliers in their respective distributions. It should be noted that the placebo test is passed regardless of whether the explanatory variable is *SPI1* or *SPI2*. For the sake of saving space, we only use Fig 1 to illustrate the results of the placebo test with *SPI1*. This suggests that our baseline estimation results are unlikely to be spurious correlations caused by omitted variables.

#### 3. PSM-DID

To avoid sample selection bias, we also construct a propensity score matched (PSM) method to select the control group the year before Google withdrawal. We select the firms’ characteristics as matching variables: *MVE*, *MTB*, *INST*, *TURN*, *BETA*, and *SKEW*. We also include industry and location fixed effects in the logit model. The sample is matched with 1:1 without replacement, and the calliper is 0.05, used to select the control group sample. To begin with, we calculate the predicted probability values for each firm in the treatment group based on observable matching variables. Subsequently, a unique control group firm is identified for each firm in the treatment group. To validate the robustness of our matching outcomes, we assess the balance assumption of score matching. The results, presented in Panel B and Panel C of Table 2 before and after matching, respectively, undergo testing. Examination of the results reveals that post-matching, the t-statistics for all variables are not significant. This indicates that, after matching, there is no noteworthy difference between the treatment and control groups regarding the matched variables. Thus, the samples obtained after matching ensure the randomization of the treatment, affirming the reliability of the estimation results presented in this paper. Columns 3 and 4 of Table 3 show the estimation results based on the PSM sample. The coefficient of TREAT*POST are both -0.055, which is statistically significant at the conventional level, indicating that our results are not influenced by sample selection bias.

#### 4. Alternative measures of the stock price informativeness

We use two alternative variables to measure the stock price informativeness, including the *R*^*2*^ obtained by regression Fama three-factor model [25] and the probability of informed trading [26]. Both of these variables are denoted *SPI3* and *PIN*, respectively. The estimation result is shown in Column 5 and Column 6 of Table 3, separately. We find that the coefficients of TREAT*POST are -0.149 in Column 5 and -0.132 in Column 6, and both coefficients are significant at least at the 5% significance level.

## 4. Cross-sectional tests

So far, we have adequately established an average causal effect of Google withdrawal on stock price informativeness. However, several factors that may render the effect of Google withdrawal heterogeneous. We conduct the cross-sectional tests along three important dimensions, namely retail investors, ownership and infomediary. By comparing the patterns of stock price informativeness in different subsamples, we endeavor to gain a deeper comprehension of the underlying mechanism for the effect of Google withdrawal on stock price informativeness.

### 4.1 Retail investors

We cannot ignore the fact that most investors in China are retail investors, who have fewer ways to obtain information but extensively use online to searches [3]. On the contrary, sophisticated institutional investors have various channels, such as field surveys [27], broker-seller services [28], etc. Therefore, the negative effect of Google withdrawal on the stock price informativeness is only significant in firms with more retail investors. We separate our sample based on the number of investors at the end of the year.

Table 4 shows the regression results grouped by the number of investors. Consistent with our conjecture, we observe that the negative effect of Google withdrawal on the stock price informativeness is significant only for firms with a higher number of retail investors. The evidence in Table 4 further supports the argument that Google withdrawal decreases the ability of retail investors to process information.

**Table 4:** Internet searching, retail investors and the stock price informativeness.

	(1)	(2)	(3)	(4)
	High retail investors	Low retail investors	High retail investors	Low retail investors
	*SPI1*	*SPI1*	*SPI2*	*SPI2*
TREAT*POST	-0.128***	-0.043	-0.117***	-0.044
	(0.040)	(0.043)	(0.038)	(0.040)
*MVE*	0.202***	0.261***	0.197***	0.248***
	(0.038)	(0.034)	(0.036)	(0.032)
*MTB*	0.058***	0.063***	0.058***	0.050***
	(0.016)	(0.011)	(0.014)	(0.011)
*INST*	0.965***	0.042	1.047***	0.006
	(0.193)	(0.214)	(0.184)	(0.193)
*TURN*	0.073***	0.086***	0.075***	0.076***
	(0.006)	(0.007)	(0.006)	(0.007)
*BETA*	-1.198***	-1.466***	-1.082***	-1.268***
	(0.067)	(0.064)	(0.061)	(0.058)
*SKEW*	0.248***	0.197***	0.202***	0.165***
	(0.028)	(0.023)	(0.027)	(0.023)
*ANALYST*	-0.012	-0.061***	-0.020	-0.053***
	(0.017)	(0.017)	(0.016)	(0.015)
*MEDIA*	0.043**	0.044**	0.030*	0.028*
	(0.018)	(0.017)	(0.017)	(0.016)
*BAIDU*	0.033***	0.012	0.025***	0.011
	(0.009)	(0.009)	(0.009)	(0.008)
*Constant*	-3.989***	-4.523***	-4.071***	-4.482***
	(0.825)	(0.742)	(0.782)	(0.680)
Firm fixed effects	*Yes*	*Yes*	*Yes*	*Yes*
Year fixed effects	*Yes*	*Yes*	*Yes*	*Yes*
N	4,675	4,680	4,675	4,680
Adj. *R*^*2*^	0.468	0.493	0.461	0.467

Notes: Standard errors are robust and clustered at the firm level

***, **, * indicate significance at the 1%, 5%, and 10% levels, respectively.

### 4.2 Ownership

Due to the absence of well-defined property rights and clear principals, politically affiliated firms are commonly regarded as having lower transparency and a poor information environment [29]. The information disclosure of such firms often needs to align with the political objectives of their jurisdictions’ government, resulting in a lack of timeliness. For instance, Piotroski et al. [30] found that during some significant central political meetings, state-owned enterprises were typically constrained from releasing negative news about themselves, leading to an increased risk of future stock price collapses. In contrast, private enterprises did not experience similar constraints on information disclosure. Thus, through internet searching, retail investors need to collect more firm-specific information to invest in state-controlled listed firms. However, the information discovery function of internet searching may be hindered after Google withdrawal. We split the whole sample into sub-samples based on whether the state controls the firms.

The results in Table 5 show that the effect of Google withdrawal on the stock price informativeness is negative and significant only for SOE firms. Therefore, our results are consistent with the view that retail investors can obtain more specific information for those firms with information opacity through internet searching.

**Table 5:** Internet searching, state control and the stock price informativeness.

	(1)	(2)	(3)	(4)
	*SOE*	*NON-SOE*	*SOE*	*NON-SOE*
	*SPI1*	*SPI1*	*SPI2*	*SPI2*
TREAT*POST	-0.093***	-0.038	-0.087***	-0.039
	(0.034)	(0.046)	(0.031)	(0.044)
*MVE*	0.215***	0.235***	0.208***	0.230***
	(0.030)	(0.039)	(0.028)	(0.037)
*MTB*	0.073***	0.057***	0.064***	0.048***
	(0.012)	(0.012)	(0.011)	(0.012)
*INST*	0.886***	0.074	0.914***	0.033
	(0.168)	(0.226)	(0.156)	(0.208)
*TURN*	0.076***	0.082***	0.074***	0.075***
	(0.006)	(0.007)	(0.006)	(0.006)
*BETA*	-1.296***	-1.440***	-1.141***	-1.272***
	(0.057)	(0.067)	(0.052)	(0.062)
*SKEW*	0.227***	0.171***	0.195***	0.131***
	(0.022)	(0.026)	(0.020)	(0.026)
*ANALYST*	-0.027*	-0.028	-0.034**	-0.019
	(0.015)	(0.019)	(0.014)	(0.017)
*MEDIA*	0.024*	0.025	0.014	0.014
	(0.015)	(0.019)	(0.014)	(0.018)
*BAIDU*	0.016**	0.011	0.011*	0.006
	(0.007)	(0.009)	(0.006)	(0.008)
*Constant*	-3.911***	-4.034***	-4.002***	-4.156***
	(0.649)	(0.841)	(0.605)	(0.791)
Firm fixed effects	*Yes*	*Yes*	*Yes*	*Yes*
Year fixed effects	*Yes*	*Yes*	*Yes*	*Yes*
N	6,092	3,709	6,092	3,709
Adj. *R*^*2*^	0.469	0.480	0.458	0.455

Notes: Standard errors are robust and clustered at the firm level

***, **, * indicate significance at the 1%, 5%, and 10% levels, respectively.

### 4.3 Infomediary

Analysts, as pivotal processors and disseminators of corporate information in the capital market, to some extent, may serve as substitutes for the information search function on the internet. For instance, analyst recommendations are crucial information source guiding investors’ investment decisions [31]. More specifically, the accuracy of analysts’ forecasts is closely linked to the transparency of firms and the quality of information disclosure [32], leading retail investors to depend heavily on analyst recommendations for decision-making. Given these considerations, we can hypothesize that the impact of Google withdrawal on the stock price informativeness is particularly pronounced in firms characterized by less accurate analyst earnings forecasts. We follow the practice of Merkley et al. [33] measured the analyst’s earnings forecast accuracy as follows:

$$AF\_accuracy_{k,i,t}=-\frac{\left|FEPS_{k,i,t}‐EPS_{i,t}\right|}{\left|Consensus\;EPS_{i,t}\right|},$$ (5)

where *AF_accuracy_k,i,t_* is the earning forecast accuracy of analyst k, for firms i at year t, the higher its value represents higher precision. *FEPS_k,i,t_* is the analyst’s forecasts of earnings per share in year t. *EPS_i,t_* is the firms’ actual earnings per share in year t. *ConsensusEPS_i,t_* is the median of forecasts of earnings per share for all analysts in year t. We calculate the mean value of *AF_accuracy_k,i,t_* for each firm and each year to obtain its value at the firm level, denoted as *AF_accuracy*.

We separate our sample using the median value of *AF_accuracy* at each year. The results in Table 6 show that the effect of Google withdrawal on the stock price informativeness is negative and significant at the conventional level only for those firms with low analysts’ earnings forecasts accuracy. Thus, we conclude that internet searching, in addition to analyst recommendations, is an essential source of information for investors.

**Table 6:** Internet searching, information transparency and the stock price informativeness.

	(1)	(2)	(3)	(4)
	High accuracy	Low accuracy	High accuracy	Low accuracy
	*SPI1*	*SPI1*	*SPI2*	*SPI2*
TREAT*POST	-0.039	-0.094**	-0.038	-0.095**
	(0.045)	(0.045)	(0.044)	(0.042)
*MVE*	0.202***	0.295***	0.222***	0.274***
	(0.043)	(0.040)	(0.041)	(0.038)
*MTB*	0.035**	0.037**	0.025	0.030**
	(0.016)	(0.017)	(0.016)	(0.015)
*INST*	0.546**	0.440**	0.505**	0.503***
	(0.214)	(0.195)	(0.200)	(0.186)
*TURN*	0.083***	0.075***	0.079***	0.071***
	(0.009)	(0.008)	(0.009)	(0.008)
*BETA*	-1.219***	-1.416***	-1.131***	-1.222***
	(0.072)	(0.077)	(0.067)	(0.069)
*SKEW*	0.201***	0.203***	0.161***	0.172***
	(0.027)	(0.033)	(0.026)	(0.031)
*ANALYST*	0.039*	-0.052***	0.031	-0.046**
	(0.022)	(0.020)	(0.021)	(0.019)
*MEDIA*	0.006	0.036*	-0.006	0.023
	(0.021)	(0.021)	(0.020)	(0.019)
*BAIDU*	0.016*	0.027***	0.012	0.026***
	(0.009)	(0.010)	(0.009)	(0.010)
*Constant*	-3.536***	-5.415***	-4.115***	-5.224***
	(0.933)	(0.852)	(0.895)	(0.807)
Firm fixed effects	*Yes*	*Yes*	*Yes*	*Yes*
Year fixed effects	*Yes*	*Yes*	*Yes*	*Yes*
N	3,791	3,802	3,791	3,802
Adj. *R*^*2*^	0.428	0.477	0.405	0.463

Notes: Standard errors are robust and clustered at the firm level

***, **, * indicate significance at the 1%, 5%, and 10% levels, respectively.

## 5. Discussion

In this section, we further elucidate the abovementioned findings, expand their theoretical and practical implications, and then point out the limitations and possible future research.

### 5.1 Interpreting findings

Understanding how and to what extent internet searching impacts stock price informativeness is not only crucial for improving investor efficiency but also intuitively important for enhancing the operational efficiency of the stock market. Previous research on determinants of stock price informativeness has primarily focused on internal governance and external supervisory environments. Scholars have found that factors such as corporate board structure, ownership characteristics, legal environment, and informal institutions are all essential determinants of stock price informativeness [13, 14, 26, 34]. Additionally, more scholars recognize the significant role of investors acquiring information in stock price informativeness, which is intuitively generated directly by investors’ stock transactions. For instance, Cheng et al. [35] found that investors conducting on-site visits to listed companies increase stock price informativeness. However, unlike institutional investors, the majority of retail investors are unlikely to acquire company-specific information through on-site visits, but internet searching is a notable exception [3]. Nevertheless, there has been no scholarly attention to whether investors’ access to firms-specific information through internet searching can impact stock price informativeness. Based on the existing theoretical foundation, we hypothesize that internet searches could enhance investors’ understanding of firm-specific information, consequently leading to a significant increase in stock price informativeness.

To address the endogeneity concern, we leverage the 2010 Google withdrawal from mainland China as a natural experiment for internet searching. Employing a difference-in-differences (DID) analysis, we examine the impact of the decline in internet searching functionality on stock price informativeness. The empirical findings reveal a significant reduction in stock price informativeness following Google withdrawal, indicating that investors use the internet to search for firm-specific information manifested in stock price changes. It should be noted that due to the absence of investor-level trading data, our study focuses on the firm level, assuming a significant correlation between the search volume of company codes and investor trading behavior. Nevertheless, our research findings represent a further extension and complement to the existing literature. To some extent, our finding is consistent with the theoretical insights presented by Han and Yang1 [36]. Using a rational expectations equilibrium model, they explored the relationship between information sources and market efficiency. The outcomes of their study suggest that social communication enhances market efficiency when information is exogenous. However, it can crowd out information production when information originates from insiders. Hence, our emphasis is on the idea that internet searching not only facilitates investors in accessing new information but also plays a role in information generation rather than mere dissemination.

We delve into three crucial aspects of heterogeneity in the impact of Google withdrawal on the stock price informativeness. Firstly, we observe that the effect of Google withdrawal is more pronounced for firms whose shareholders are predominantly retail investors. This outcome enhances the causal inference of our primary result, given that retail investors are more inclined to rely on the internet for stock market information acquisition [37]. In contrast, institutional investors, with their specialized teams and scale advantage, can procure firms’ operational information through alternative means [27, 28]. Consequently, compared to institutional investors, the adverse impact of Google withdrawal on information accessibility might be more substantial for retail investors. Besides, our study complements the work of Xu et al. [3], who find that the positive effect of Google withdrawal on stock price crash risk is more prominent among firms with more retail investors. Meanwhile, this can also be explained by diminished effective accessibility of firms’ information among retail investors resulting from Google withdrawal.

The results of the second heterogeneity analysis indicate that the detrimental impact of Google withdrawal on the stock price informativeness is particularly pronounced among state-controlled firms. State-owned enterprises (SOEs) are commonly perceived to possess deficient corporate governance structures and business objectives influenced by political considerations [30]. Consequently, they often fall short in providing sufficient firm-specific information, such as voluntary disclosures, to meet the informational needs of investors. Despite previous research pointing out distinctions in information transparency between state-owned and non-state-owned firms [29, 38]. In line with their ideas, our findings suggest that the adverse effect of the Google withdrawal on the stock price informativeness is enhanced mainly due to the lower information transparency of state-owned firms. Once again, it implies that the firms’ nature of equity is an important boundary condition for Google withdrawal to reduce the stock price informativeness.

Another notable finding in our heterogeneity analysis is the crucial moderating role of information intermediaries in the impact of Google withdrawal on the stock price informativeness. Using analysts as a key proxy for information intermediaries, our results indicate that the higher the accuracy of analysts’ earnings forecasts, the less significant the effect of Google withdrawal on the stock price informativeness. The results clearly illustrates a discernible substitution between internet searching and analysts regarding investors’ access to information. Existing perspectives on the relationship between analysts and other information intermediaries, often called as media, are inconclusive. Some suggest complementarity [39], some propose the latter as an informational emulator of the former [40], and others argue for a substitutability relationship between the two [41]. Our findings align with the latter perspective, indicating that internet searching acts as a substitute for the information brokering function of analysts.

Finally, in contemplating the applicability of our results beyond China, it is essential to consider the diverse market environments and regulatory landscapes that characterize global financial systems. Our study, rooted in the unique circumstances of the 2010 Google withdrawal in China, prompts interesting reflections on the potential generalizability of the observed causal relationship between internet searching and stock price informativeness. In economies with varying market dynamics, the impact of internet searching on stock price informativeness could be nuanced. Factors such as the degree of market competition, information transparency, and regulatory frameworks may play pivotal roles in shaping the observed patterns. Additionally, the interplay between internet censorship and information dissemination channels could lead to divergent outcomes.

### 5.2 Implications

Our study deepens the understanding that investors can utilize internet search services to obtain valuable information, thereby enhancing stock price informativeness. At this point, our study holds several implications. Firstly, our work contributes to stakeholder and information asymmetry theories. By investigating the impact of internet searching on stock price informativeness through the lens of Google withdrawal from mainland China, our findings introduce a new dimension to the explanation of stock price informativeness. Secondly, examining behavioral differences between individual and institutional investors is widely debated [42–44]. Our results indicate that the negative effect of Google withdrawal on the stock price informativeness is significant only when the number of retail investors is high. This insight contributes to our understanding that a crucial aspect of the behavioral differences between individual and institutional investors lies in the way of information acquisition; individual investors primarily obtain firm-specific information from the internet, whereas institutional investors do not.

The research presented in this paper holds significant implications for government regulation and policy formulation. China’s A-share market ranked second in total market capitalization globally after the U.S. stock market but underperformed compared to other major stock markets [45]. An essential factor contributing to the inefficiency of A-share investments is the limited access investors have to comprehensive information about listed companies. Despite alternative search engines such as Baidu and Sogou being available post-Google withdrawal, our findings reveal that firms with higher Google search volumes experienced a substantial decrease in stock price informativeness. Then, we can assert that other search engines are ineffective substitutes for Google. To tackle this challenge, the Chinese authorities have the opportunity to bolster oversight of internet search platforms and optimize information amalgamation, ensuring the furnished data is genuinely beneficial for investors. Furthermore, regulatory bodies can enact pertinent statutes and protocols to incentivize either mandatory or voluntary disclosures by publicly listed entities across various online platforms. Given the distinctive landscape of China’s A-share market, predominantly steered by retail investors and marked by robust speculative sentiments, concerted efforts from the government or relevant entities are essential. These efforts should include fortified initiatives in investor education to enhance financial literacy. Empowering retail investors with the ability to adeptly discern authentic information from misinformation regarding listed companies on the Internet is paramount. Guiding investors towards a value-based investment approach can, in turn, significantly enhance market efficiency. These initiatives aims to enhance transparency and the quality of information disclosure, empowering investors to assess the value and risks associated with a company more accurately.

Additionally, our findings carry significant managerial implications for firms. Previous research has consistently demonstrated that stock price informativeness is positively associated with firms’ external governance and productivity [1, 4, 46]. Therefore, improving stock price informativeness stands as a crucial practical concern for corporate governance. Our findings show that internet searching substantially enhances stock price informativeness, particularly in firms with a substantial number of retail investors. As a practical approach, companies can enhance transparency and the quality of information disclosure by providing more accurate and valuable information online. Furthermore, our study reveals that the adverse impact of Google withdrawal on the stock price informativeness is predominantly observed among state-owned companies, suggesting a lack of initiative in providing sufficient information to investors. In response, state-owned firms should strategically optimize their corporate governance structure and prioritize regular communication with investors, especially retail investors. These proactive measures empower retail investors to make more informed stock trading decisions.

### 5.3 Limitations and future research

Our work leaves several areas of research deficiencies that could be explored in depth in future research. Firstly, the absence of investor-level trading data in our current dataset limits our ability to investigate whether the trading behaviors of all investors were uniformly influenced by the movement of the composite index post-Google’s withdrawal. Future research could use finer-grained investor trading data to dissect the impact of internet searching on investor behavior. Second, we do not delve into whether internet searching could influence firm behavior. Specifically, investors gaining access to new information through internet searching might impact their stock transactions, creating a more effective external monitoring mechanism for firms and enhancing operational efficiency. Therefore, we can further extend the analysis to examine the impact of internet searching on corporate financial behavior, shareholder actions, and stock systemic risk. As these aspects are crucial for both the businesses of firms and their value [47–50]. Lastly, our study concentrates on the role of internet searching in stock price informativeness without fully considering both direct and indirect effects. Previous research has indicated that Google withdrawal results in a substantial decline in firm innovation [51] and a decrease in the quality of export products [52], suggesting that the Google withdrawal event may also influence firms’ disclosure behavior. Future research could explore whether Google’s withdrawal indirectly affects the stock price informativeness by influencing firm behavior.

## 6. Conclusion

We investigated the implications of stock price informativeness of internet searching using a representative sample of Chinese companies. To identify causality, we conduct a difference-in-differences analysis to explore the effect of Google sudden withdrawal from mainland China in 2010 on the stock price informativeness. Our empirical results show firms with a high initial Google search volume whose stock price informativeness decreased about 10% after Google withdrawal. Our results remain consistent across various robustness checks. Further analysis implies that the effect is greater in firms with more retail investors, larger state-ownership, and less favourable analysts’ earning forecast accuracy. Overall, our findings are consistent with the hypothesis that retail investors cannot effectively gather firms-specific information after Google withdrawal, thus making the stock market less efficient.

## Supporting information

S1 Appendix Contains supporting tables. (DOCX)

S1 Data (ZIP)
