# Supplementary material for: Internet searching and stock price informativeness: Evidence from Google withdrawal in China
Source: PLoS One. 2024 Mar 13;19(3):e0297160. doi: 10.1371/journal.pone.0297160 (PMC10936803; doi:10.1371/journal.pone.0297160)
Supplement: S1 Appendix — (DOCX) [file pone.0297160.s001.docx]

## Appendix

**Table A1. Definition of key variables**

| Variable | Definition |
| --- | --- |
| *SPI1* | Stock price informativeness. *SPI1= ln[(1-R^2^)/R^2^)]*, where *R^2^* is the R-squared value obtained from estimating the model according to Morck et al. [10] using weekly return data. |
| *SPI2* | Stock price informativeness . *SPI1= ln[(1-R^2^)/R^2^)]*, where *R^2^* is the R-squared value obtained from estimating the model according to Piotroski et al. [11] using weekly return data. |
| *TREAT* | Dummy variable that equals 1 for firms whose stock tickers have a higher Google search volume index than the sample median in 2009, and 0 otherwise. |
| *POST* | Dummy variable that equals 1 for firms in 2010-2014, and 0 otherwise. |
| *MVE* | Natural logarithm of the firms’ market value. |
| *MTB* | Market value divided by book value of equity. |
| *INST* | Number of shares held by institutional investors divided by total number of shares outstanding. |
| *TURN* | Monthly stock trading volume divided by total number of shares outstanding at the end of the month, then take the annual average of this ratio. |
| *BETA* | Market beta of monthly stock returns for the year-end. |
| *SKEW* | Skewness of daily stock returns for the year. |
| *ANALYST* | Natural logarithm of the number of analyst plus 1. |
| *MEDIA* | Natural logarithm of the number of news released by media plus 1. |
| *BAIDU* | Natural logarithm of the Baidu search index plus 1. |

**Table A2. Propensity score matching**

| *Panel A: Propensity score logit regression for the treatment and control groups* | | | | |
| --- | --- | --- | --- | --- |
|  | *TREAT* | | | |
| *MVE* | 0.423*** | | | |
|  | (0.105) | | | |
| *MTB* | 0.096* | | | |
|  | (0.049) | | | |
| *INST* | 2.017** | | | |
|  | (0.855) | | | |
| *TURN* | 0.196*** | | | |
|  | (0.032) | | | |
| *BETA* | -0.518 | | | |
|  | (0.464) | | | |
| *SKEW* | 0.849*** | | | |
|  | (0.287) | | | |
| Industry fixed effects | Yes | | | |
| Province fixed effects | Yes | | | |
| N | 1203 | | | |
| Pseudo R^2^ | 0.109 | | | |
| χ^2^ | 181.53 | | | |
| p-value of χ^2^ | 0.000 | | | |
| *Panel B: Covariate Balance test for pre-matching sample* | | | | |
|  | Mean | | t-test | |
| Variable | Treated | Control | T-value | P-value |
| *MVE* | 22.495 | 22.344 | 2.780 | 0.005 |
| *MTB* | 2.663 | 2.393 | 3.070 | 0.002 |
| *INST* | 0.092 | 0.063 | 5.260 | 0.000 |
| *TURN* | 5.934 | 5.399 | 3.370 | 0.001 |
| *BETA* | 1.066 | 1.082 | -1.360 | 0.175 |
| *SKEW* | -0.007 | -0.073 | 4.320 | 0.000 |
| *Panel C: Covariate Balance test for post-matching sample* | | | | |
|  | Mean | | t-test | |
| Variable | Treated | Control | T-value | P-value |
| *MVE* | 22.386 | 22.379 | 0.110 | 0.912 |
| *MTB* | 2.397 | 2.479 | -0.810 | 0.419 |
| *INST* | 0.071 | 0.070 | 0.160 | 0.872 |
| *TURN* | 5.498 | 5.662 | -0.870 | 0.384 |
| *BETA* | 1.078 | 1.073 | 0.360 | 0.720 |
| *SKEW* | -0.051 | -0.041 | -0.570 | 0.570 |

Notes: Panel A is the estimation results using a the logit model. ***, **, * indicate significance at the 1%, 5%, and 10% levels, respectively.
